# Surgical correction of proximal junctional kyphosis

**DOI:** 10.3171/2020.1.FocusVid.19733

**Published:** 2020-01-01

**Authors:** Jamal McClendon, Richard Shindell, Karl R. Abi-Aad, Ahmad Kareem Almekkawi, Tanmoy Maiti, Bernard R. Bendok

**Affiliations:** 1 Department of Neurological Surgery,; 2 Neurosurgery Simulation and Innovation Lab,; 3 Precision Neuro-therapeutics Innovation Lab,; 4 Department of Otolaryngology, and; 5 Department of Radiology, Mayo Clinic, Phoenix; and; 6 Department of Orthopedic Surgery, Phoenix Children’s Hospital, Phoenix, Arizona

**Keywords:** junctional kyphosis, correction, individualized approach, video

## Abstract

This 3D video showcases the surgical techniques for patients with proximal junctional kyphosis. The surgical repair for patients with proximal junctional kyphosis is an individualized approach depending on patient history and imaging with adequate surgical measurements. This video will shed light on two cases with proximal junctional kyphosis and the method taken for their repair. The first case is of an 11-year-old female known to have osteogenesis imperfecta and status post T5–L3 posterior spinal fusion with segmental instrumentation. The patient underwent change of older instruments and scoliosis repair, with full correction on postoperative x-ray. The second patient is a 16-year-old male known to have cerebral palsy and kyphoscoliosis status post spinal fusion. The patient underwent scoliosis repair surgery with replacement of old instrumentation and scoliosis correction.

The video can be found here: https://youtu.be/f5iLwqbU26Q.

## Transcript

In this surgical video, we present two pediatric cases where two different surgical techniques were performed to achieve comparable correction. The first patient is an 11-year-old girl with history of bony dysplasia and osteogenesis imperfecta. She underwent T5–L3 posterior instrumentation 3 years back by another surgeon. Unfortunately, she developed proximal junctional kyphosis, which worsened over time.

Various measurements showed sagittal malalignment and proximal junctional kyphosis was confirmed radiologically. In this surgical video we see a Gardner-Wells traction was applied at neutral position about 3 cm above the external auditory meatus. Intraoperative neuromonitoring was implemented. Patient was flipped to prone position in a Jackson table; 15-pound weight was attached to the traction.

Subsequently, somatosensory and motor evoked potential recording were obtained before and after the flipping use of a cell saver was implemented. Subsequently, we proceeded with the opening of the previous incision, and we extended the incision slightly rostrally. Solid fusion was noted in previous level. Bilateral facet joints and lamina were exposed. Bilateral inferior facetectomy was done using bone scalpel half-inch and quarter-inch osteotome and mallet. Pituitary forceps and rongeur were used to collect the bone pieces, which were later used as autograph.

At this point, we were open to both options, which is posterior column osteotomy at multiple levels, versus vertebral column rejection. However, considering the fact that patient had osteogenesis imperfecta we started with posterior column osteotomy, caudal to cranial. Wide-symmetry posterior column osteotomy using Kerrison punch, 3 and 2. We noted that spine became gradually mobile, allowing us to achieve the correction.

Subsequently we placed the pedicle screws, from T1 to T4, using Lenke’s freehand technique. The screw entry point is slightly different from T4 to T1, as depicted here. Rostral screws were increasingly difficult because of the angulation. A 4.5-mm tap was used before placing 5.5-mm screws. We also upsized the screws at T5 and T6 level. The facet joints above the rostral-most screws were preserved, to prevent degeneration on a later date. Finally, one side rod was removed and a new rod was placed. Same steps were repeated on the other side.

As we know, both rods were never removed at the same time. Cantilever technique was used for rod placement and compression was applied on each level to close the osteotomy. Neuromonitoring was stable throughout the surgery and postop x-ray revealed complete correction of the sagittal malalignment.

The second patient is a 16-year-old boy with a history of cerebral palsy, secondary to anoxic brain injury. He also developed thoracolumbar scoliosis. He underwent T4–L3 fusion 2 years back by another surgeon. Unfortunately, he developed proximal junction kyphosis, which worsened with time. Here you can see the various measurements in sagittal and coronal plane before our surgery. During the surgery, Gardner-Wells traction was placed, previous instruments were removed and exchanged. The instrumentation was extended rostrally to include bilateral T1, T2, and T3 pedicles. Vertebral column resection was performed at T4 level, and custom Lenke vertebral body cage was placed. We could achieve a good correction in sagittal as well as caudal planes at the end of the surgery, which was confirmed by the x-ray.

In this video, we discussed two patients with proximal junctional kyphosis. In the first patient bone quality was a concern, as he has osteogenesis imperfecta. We could achieve good correction, after multiple-level wide posterior column osteotomy. For the second patient, multilevel PCO was not enough so we had to proceed for vertebral column resection.

The treatment is mostly individualized. The surgeon has to be flexible and plan according to the situation. And a team of two experienced spine surgeons can reduce the operative time and complication and improve the expertise.

### Time points

0:49 Preoperative imaging depicting proximal junctional kyphosis

2:17 Lines depicting levels of the multilevel posterior column osteotomy

4:03 Postoperative imaging showing correction of the kyphosis

4:36 Steps of proximal junctional kyphosis correction

## Acknowledgments

We would like to thank Michael A. King for his contribution to the medical illustrations presented in this surgical video.
